# Risk of Uterine Rupture with Vaginal Birth after Cesarean in Twin Gestations

**DOI:** 10.1155/2021/6693142

**Published:** 2021-03-31

**Authors:** Kimya Baradaran

**Affiliations:** Master of Science in Physician Assistant Studies, Dominican University of California, San Rafael, CA 94901, USA

## Abstract

**Background:**

Women with a previous cesarean delivery may attempt a subsequent vaginal birth or repeat cesarean. Vaginal birth after cesarean carries a greater risk of uterine rupture, defined as the disruption of all uterine layers, resulting in maternal-fetal morbidity or mortality. It is unclear how the risk of uterine rupture compares in patients with twin gestations who undergo different delivery methods.

**Objective:**

The purpose of this systematic review is to determine if there is an increased risk of uterine rupture in patients with twin gestations attempting vaginal birth after cesarean (VBAC) versus planned repeat cesarean delivery (PRCD). *Study Design*. PubMed, Cochrane Library, and CINAHL were searched systematically. Eligible studies were prospective and retrospective studies that evaluated the incidence of uterine rupture in twin pregnancies that attempted VBAC or PRCD. Data were manually extracted from these studies, and the number of events in each group was used to calculate an odds ratio (OR) and 95% confidence interval (CI).

**Results:**

4 retrospective studies were included with a total of 7699 participants, 2305 of whom attempted VBAC and 5394 underwent PRCD. The absolute risk of uterine rupture in the VBAC and PRCD groups was 0.87% and 0.09%, respectively. The rate of uterine rupture was significantly higher in the VBAC group than in the PRCD group (OR: 9.43; CI: 3.54–25.17).

**Conclusion:**

Although VBAC is associated with higher rates of uterine rupture in twin pregnancies when compared with PRCD, the absolute risk of uterine rupture is low in both groups. Depending on individual risk factors, vaginal birth may be offered as a safe option to women with twin pregnancies and a history of cesarean delivery.

## 1. Introduction

The rate of cesarean deliveries in the United States has increased significantly, from 5.5% in 1970 to 31.9% in 2016 [1]. Similarly, the national rate of twin deliveries has increased 79% from 1980 to 2016 (from 18.9 to 33.9 per 1,000) [2]. As a result, obstetricians are encountering more patients with both twin gestations and a history of cesarean. Multiple pregnancies (97–98% of which are twins) have a twofold risk of maternal death and more complications including eclampsia, postpartum hemorrhage, and preterm labor [3–5]. Women with a previous cesarean have a greater risk of placental issues and twice the risk of maternal morbidity, which increases progressively as the number of previous cesareans increases [6–10].

Women with a previous cesarean have the option of attempting vaginal birth after cesarean (VBAC) or a planned repeat cesarean delivery (PRCD) in a subsequent pregnancy [11]. Overall, 73.6% of VBAC attempts result in a successful vaginal delivery [12]. The likelihood of achieving VBAC varies based on demographic and obstetric characteristics. Risk factors for failed VBAC include maternal age ≥35, maternal body mass index ≥30, birth weight >4000 g, and gestational age >40 weeks at delivery [12–16]. Evidence also shows that labor induction or augmentation with oxytocin reduces the chance of a successful VBAC when compared to spontaneous labor without augmentation [12]. Compared to PRCD, VBAC attempts have higher rates of endometritis, respiratory distress syndrome, and uterine rupture, but lower rates of hysterectomy and wound complications, shorter recovery periods, and less blood loss [11, 17–19].

Uterine rupture is defined as a complete disruption of all uterine layers, including the serosa, resulting in a change in maternal or fetal status [20]. The incidence of uterine rupture is 0.4–0.7% in patients who attempt VBAC, but this risk is higher with increased maternal and gestational age and induction with oxytocin [21–25]. Fetal complications of uterine rupture include hypoxic-ischemic encephalopathy, impaired motor development, and death, while maternal complications include postpartum hemorrhage, hysterectomy, genitourinary injury, and death [21, 22, 24, 26]. Evidently, uterine rupture, while rare, carries a high risk of maternal-fetal morbidity and mortality. Various studies have shown null and positive associations between VBAC attempts in twin pregnancies and the risk of uterine rupture as compared to PRCD [27–34]. Due to this controversy in the literature, there is a need for a systematic review. This systematic review aims to determine the risk of uterine rupture with VBAC attempts versus PRCD in patients with twin gestations. The results of this study will aid in clinical decision-making when recommending patients with a history of cesarean to deliver twins vaginally versus via planned cesarean.

## 2. Methods

The current systematic review was conducted in accordance with the Preferred Reporting Items for Systematic Reviews and Meta-Analysis (PRISMA) guidelines. A systematic manual search of major databases was conducted in PubMed, Cochrane Library, and CINAHL to identify all prospective observational studies and retrospective cohort studies comparing VBAC attempt and PRCD in twin gestations. The search was completed from inception to September 2018 without any language restrictions. The PICO (patient, intervention, comparator, and outcome) statement was used to perform the literature search. Search terms were related to the population of interest (women with twin pregnancies and a previous cesarean), intervention (VBAC attempt), comparator (PRCD), and outcome (uterine rupture). The following keywords were used: twin, trial of labor, vaginal birth after cesarean, previous cesarean, and repeat cesarean. The references of the included studies and prior reviews on the same topic were also screened to identify additional relevant articles. A stepwise approach was utilized for selecting the final studies.

All records were manually screened by title and abstract to ensure that they aligned with the population, exposure, and outcome of this study. Studies that were potential candidates were further evaluated using the inclusion and exclusion criteria. A study was included if it was prospective or retrospective, considered women with twin pregnancies and a previous cesarean, compared VBAC attempt and PRCD, and assessed for uterine rupture. Studies were excluded if they were reviews, commentaries, or case reports, not written in English, did not report any cases of uterine rupture in either group, or reported uterine dehiscence. Lastly, the qualities of the potential studies were rated using the Newcastle–Ottawa Scale (NOS). Studies were included if they received a fair or good rating, but were eliminated if they received a poor rating (defined as 0–1 stars in the selection domain, 0 stars in the compatibility domain, or 0–1 stars in the exposure/outcome domain). Clinically relevant data were extracted regarding study year, study design, and study period, single versus multicenter study, total number of participants, number of participants in each group, and number of events in each group. Since the outcome was not present in every group, the sum of the events across the studies was used to calculate an odds ratio (OR) and 95% confidence interval (CI).

## 3. Results

**Figure 1:**
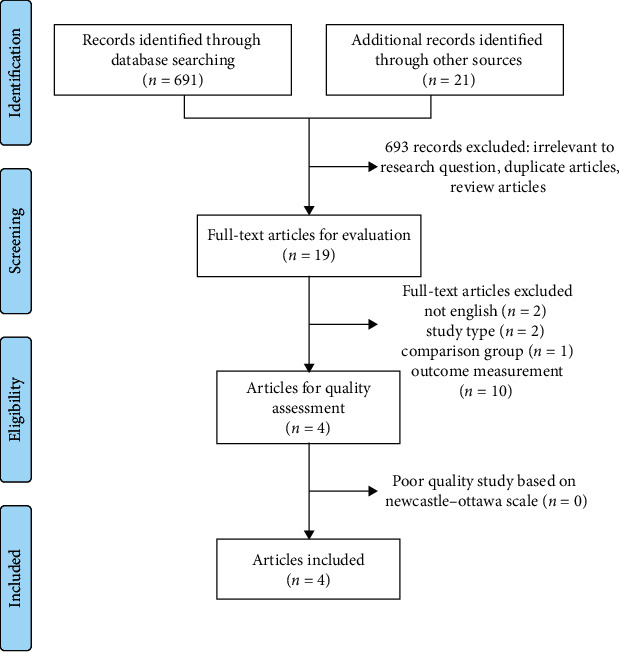
Study selection process.

A total of 712 records were screened and 19 full-text articles were evaluated. 15 studies were eliminated based on exclusion criteria. 4 studies were considered potentially eligible, and none of them were determined to be of poor quality (Figure 1).

A total of 4 retrospective studies originating from the United States and published between 1996 and 2006 were included in the systematic review [31–34]. 3 of the studies gathered data from multiple medical centers [32–34], while 1 study obtained data from a single hospital [31]. Collectively, the studies identified 7699 women with twin pregnancies and a previous cesarean. Of these, 2305 attempted VBAC and 5394 underwent PRCD (Table 1).

Uterine rupture rates ranged from 0% to 1.69% (Table 2). Of the 4 studies, 3 found no significant difference in uterine rupture rates between the groups [31–33], while the largest study reported an increased rate of uterine rupture with VBAC attempts [34].

## 4. Discussion

Systematic review of published studies revealed that the risk of uterine rupture is significantly higher in women with twin gestations who attempt VBAC as opposed to PRCD. However, the absolute risk of uterine rupture is low in both groups, as shown by the low percentages of uterine rupture (Table 2) and the fact that 3 out of 4 studies contained a group with 0 cases of uterine rupture [31–33]. Notably, the study with the largest patient population reported cases of uterine rupture in both groups and demonstrated a significantly greater risk of uterine rupture in the VBAC group [34]. Meanwhile, the other 3 studies found no significant difference between rates of uterine rupture among the groups [31–33]. Nevertheless, this study shows that electing to have a PRCD reduces but does not eliminate the small risk of uterine rupture.

## 5. Conclusion

This review determined that women with twin gestations and a previous cesarean delivery are at a higher risk of uterine rupture from vaginal delivery versus another cesarean. This information should be provided during prenatal counseling to help guide clinical decisions. Despite this, clinicians should be cautious not to develop an overall perception of high risk regarding VBAC in twin pregnancies. Clinicians should consider that the increased risk of uterine rupture is statistically but not necessarily clinically significant. Clinicians should discuss with their patients the option of attempting a VBAC, especially if the patient is free of additional risk factors that increase the rate of uterine rupture.

Since the data for this systematic review were obtained from multicenter studies, the results can be generalized to a broad patient population. Nevertheless, this study has some inherent limitations. First, there exists the possibility of selection bias since retrospective studies were used. Second, the number of previous cesarean or vaginal deliveries was not considered in this study, so the results may not be validated for those with a history of multiple cesareans or no prior vaginal deliveries. Last, this study focused on the risk of uterine rupture and did not consider other adverse maternal and fetal outcomes such as hemorrhage or infection, which can alter the risk-benefit ratio of each situation.

In conclusion, while the relative risk of uterine rupture is higher for VBAC attempts, the absolute risk is low so VBAC may be considered a safe and effective option in many women with twins. An individualized approach must be used to consider other risk factors, such as maternal and gestational age, that may affect the outcome of delivery. Clinicians must also consider and discuss maternal-fetal risks other than uterine rupture when determining the safest delivery method for a patient. Thus, the option of VBAC may be safely offered to women with twin gestations and a history of cesarean depending on their additional risk factors.

## Figures and Tables

**Table 1:** Baseline study characteristics [31–34].

Study and year	Study design	Study period	Single center or multicenter	Number of patients	Number of VBAC	Number of PRCD
Miller et al., 1996	Retrospective	1985–1994	Single center	210	92	118
Cahill et al., 2005	Retrospective	1996–2000	Multicenter	522	177	345
Varner et al., 2005	Retrospective	1999–2002	Multicenter	412	186	226
Ford et al., 2006	Retrospective	1993–2002	Multicenter	6555	1850	4705
Total				7699	2305	5394

**Table 2:** Study outcomes [31–34].

Study	VBAC attempt			PRCD			OR, 95% CI
	Events	Total	Percent	Events	Total	Percent	
Miller et al. [31]	0	92	0	2	118	1.69	
Cahill et al. [32]	2	177	1.13	0	345	0	
Varner et al. [33]	2	186	1.08	0	226	0	
Ford et al. [34]	16	1850	0.86	3	4705	0.06	
Total	20	2305	0.87	5	5394	0.09	9.43, [3.54–25.17]

## Data Availability

The data used to support the findings of this study are included within the article.
